# Reliability of visual assessment by non-expert nuclear medicine physicians and appropriateness of indications of [^123^I]FP-CIT SPECT imaging by neurologists in patients with early drug-naive Parkinson’s disease

**DOI:** 10.1186/s13550-019-0537-2

**Published:** 2019-07-24

**Authors:** Sven R. Suwijn, Constant V. M. Verschuur, Marleen A. Slim, Jan Booij, Rob M. A. de Bie

**Affiliations:** 1 Department of Neurology, Amsterdam University Medical Centers, University of Amsterdam, Meibergdreef 9, PO Box 22660, 1100 DD Amsterdam, The Netherlands; 2 Department of Neurology, Albert Schweitzer Hospital, PO BOX 444, 3300 AK Dordrecht, The Netherlands; 3 Department of Radiology and Nuclear Medicine, Amsterdam University Medical Centers, University of Amsterdam, Meibergdreef 9, PO Box 22660, 1100DD Amsterdam, The Netherlands

**Keywords:** DAT SPECT, Visual assessment, Indication, Reliability

## Abstract

**Purpose:**

To determine the reliability of visual assessment of [^123^I]FP-CIT SPECT imaging by non-experts in dopamine transporter (DAT) SPECT imaging in patients with early drug-naive Parkinson’s disease (PD). Also, we explored the indications of DAT SPECT imaging in clinical practice by neurologists.

**Methods:**

We collected [^123^I]FP-CIT SPECT scans of the Levodopa in EArly Parkinson’s disease (LEAP) trial participants that were made prior to recruitment, as part of routine clinical work-up. All scans were reassessed by an expert in DAT imaging. A survey on the use of DAT SPECT imaging was sent to all referring neurologists.

**Results:**

The concordance of the initial local assessment and the expert reassessment was 98.7%. The survey showed that neurologists requested DAT SPECT imaging in only 73.6% of patients to differentiate between a neurodegenerative disease and non-neurodegenerative parkinsonism.

**Conclusions:**

Visual assessment of [^123^I]FP-CIT SPECT imaging by community nuclear medicine physicians in patients with early PD is reliable. Neurologists who request DAT SPECT scans are not always aware that the high accuracy is limited only to the differentiation between neurodegenerative and non-neurodegenerative parkinsonism. A significant portion of neurologists who request DAT SPECT scans is not always aware that the high accuracy is limited to the differentiation between neurodegenerative and non-neurodegenerative parkinsonism as DAT SPECT cannot reliably distinguish the various Parkinsonian syndromes.

## Introduction

Parkinsonism is characterized by bradykinesia accompanied by either rigidity and rest tremor, or both. Parkinsonism can be classified into two clinically relevant categories: parkinsonism with nigrostriatal degeneration and parkinsonism or mimics with an intact nigrostriatal system.

First, the most frequent cause of parkinsonism with nigrostriatal cell loss is Parkinson’s disease (PD). Other, less common forms of neurodegenerative parkinsonism, also called atypical Parkinsonian syndromes (APS), include multiple system atrophy, progressive supranuclear palsy, dementia with Lewy bodies, and corticobasal degeneration.

Second, non-neurodegenerative forms of parkinsonism are disorders that are clinically established forms of parkinsonism/parkinsonism mimics but molecular imaging or autopsy shows no signs of nigrostriatal cell loss. Parkinsonism or mimics with an intact nigrostriatal system can be caused by for example medication, functional neurological symptoms, and essential tremor [1].

An accurate clinical diagnosis of PD can be challenging and misdiagnosis is not uncommon, particularly early in the course of parkinsonism since clinical features overlap frequently [2]. Distinguishing neurodegenerative versus non-neurodegenerative forms of parkinsonism is important, considering the differences in prognosis and treatment. Neuroimaging can be a useful tool to distinguish between these two categories. Dopamine transporter single-photon emission computed tomography (DAT SPECT) imaging is an accurate method to differentiate between neurodegenerative and non-neurodegenerative parkinsonism, even at an early stage of the disease [3]. The alterations of the presynaptic dopaminergic neurons can be quite similar between PD and APS. Consequently, a DAT SPECT cannot differentiate between the different forms of neurodegenerative parkinsonism in clinical practice [4].

In clinical practice, DAT SPECT images are assessed by nuclear medicine physicians, radiologists, or neurologists. Previous studies have shown that expertise in DAT SPECT imaging is preferred to visually assess DAT SPECT scans [5]. The focus of DAT SPECT imaging-related research has been on the diagnostic accuracy and the use of DAT SPECT imaging in tertiary referral centers specialized in movement disorders. Little is known about the accuracy of its use in routine clinical practice in general hospitals.

This study evaluates the reliability of visual assessments by community nuclear medicine physicians of ^123^I-2β-carbomethoxy-3β-(4-iodophenyl)-N-(3-fluoropropyl) nortropane ([^123^I]FP-CIT) SPECT imaging of patients that took part in the Levodopa in EArly Parkinson’s disease (LEAP) cohort and underwent DAT SPECT imaging prior to recruitment, as part of routine clinical practice. In addition, we present the results of a questionnaire, filled out by general neurologists, on the reasons to request DAT SPECT imaging by general neurologists.

## Methods

### Design

The current study is a retrospective cross-sectional cohort study of visual assessment of DAT SPECT scans of patients suspected to have PD that underwent [^123^I]FP-CIT SPECT imaging before they were recruited in the LEAP study. The LEAP study was a multicenter, double-blind, placebo-controlled, randomized delayed-start trial. The aim of the LEAP study was to investigate if early treatment with levodopa has a disease-modifying effect [6].

### Study population

For the LEAP study, patients in the Netherlands with recently diagnosed idiopathic PD using the standard clinical criteria were eligible [7]. The other inclusion criteria were a diagnosis made in the past 2 years, age 30 years and older, a life expectancy of more than 2 years, and no limitations in functional health for which the patient needed PD medication.

### Study procedures

For all LEAP study participants, we determined whether they underwent [^123^I]FP-CIT SPECT imaging prior to inclusion as part of routine clinical work-up. We attempted to obtain all [^123^I]FP-CIT SPECT scans. All institutions acquired and reconstructed the images according to guidelines on DAT SPECT imaging published by the European Association of Nuclear Medicine [8]. Nuclear medicine physicians without extensive expertise in DAT SPECT imaging (non-experts) in the referral hospitals assessed the scans, and [^123^I]FP-CIT SPECT scans that were evaluated by visual assessment only were included. Subsequently, the [^123^I]FP-CIT SPECT scans were visually reassessed by an expert DAT SPECT reader (JB). The expert assessor was blinded for the results of the initial assessment by the “non-expert” and clinical details aside from the date of birth and that the patients were participating in the LEAP study. The images were analyzed in a familiar and consistent color scale on a HERMES workstation.

### Classification and outcome of [^123^I]FP-CIT SPECT

**Fig. 1:**
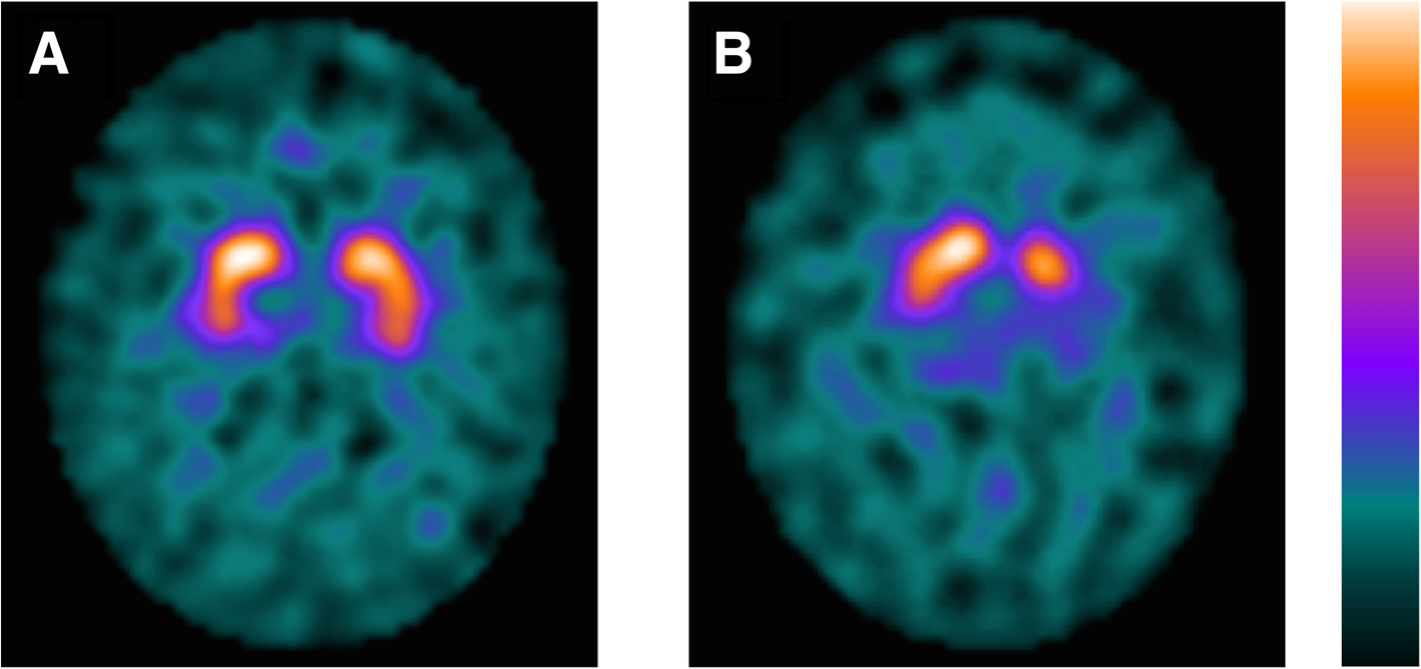
DAT SPECT imaging. Normal (**a**) and abnormal (**b**) [^123^I]FP-CIT SPECT imaging of patients in the LEAP cohort. Patient A is a 64-year-old male. Patient B is a 63-year-old female. DAT, dopamine transporter; SPECT; single-photon emission computed tomography; LEAP, Levodopa in EArly Parkinson’s disease

The [^123^I]FP-CIT SPECT images were classified as either “normal” or “abnormal.” “Normal” DAT SPECT imaging was characterized by clear binding of the radiotracer in the putamen and caudate nuclei both bilaterally, mostly symmetrical. The striata often looks circular or oval-shaped (Fig. 1) [9]. The result of DAT SPECT imaging was considered “abnormal” when low binding was visual in the striatal area, in most cases asymmetrical and lower in the putamen than the caudate nucleus. Reduced binding of the radiotracer is in the early phase of PD usually visible in the dorsal putamen and expands gradually to the ventral putamen and caudate nucleus [9].

### Use of DAT SPECT imaging in clinical practice survey

**Fig. 2:**
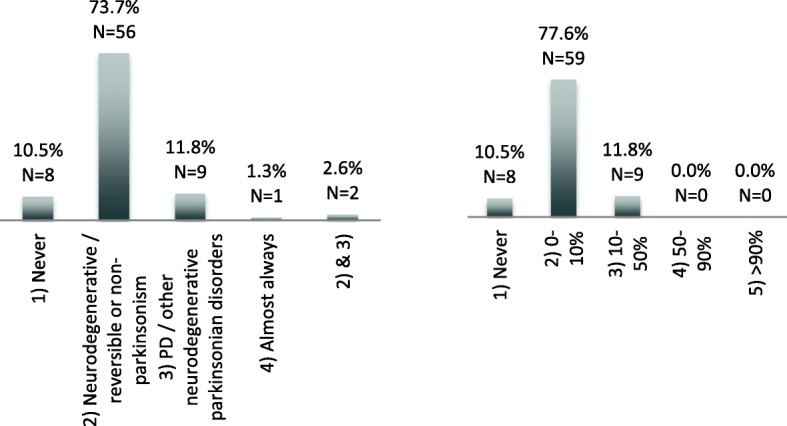
Results of the “Request of DAT SPECT imaging” survey. (1) In which case do you request DAT SPECT imaging? (left panel). (2) How often do you request DAT SPECT imaging? (right panel). DAT, dopamine transporter; SPECT, single-photon emission computed tomography; PD, Parkinson’s disease; N, number of respondents

In November 2016, an electronic survey was sent to all neurologists that referred patients for the LEAP study (*n* = 146). The questionnaire consisted of two multiple-choice questions (Fig. 2). The questions were as follows: (1) “When do you request DAT SPECT imaging?” and (2) “In which percentage of patients with a diagnosis of PD do you use DAT SPECT imaging?” The survey was completed anonymously.

## Results

### Demographics

**Fig. 3:**
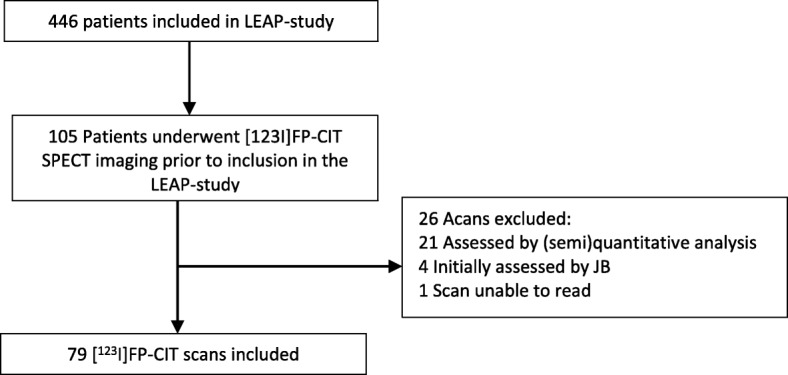
Flowchart of included DAT SPECT scans. LEAP, Levodopa in EArly Parkinson’s disease; DAT, dopamine transporter; SPECT, single-photon emission; computed tomography; JB, J. Booij

From October 2011 to May 2016, a total of 446 patients were enrolled in the LEAP study. One hundred five of these 446 patients underwent [^123^I]FP-CIT SPECT imaging prior to inclusion in the LEAP study. Twenty-six scans were excluded. Twenty-one scans were initially assessed by (semi-)quantitative analysis. Four scans were initially assessed by JB. One scan could not be reassessed due to an outdated computer system in one of the referring hospitals. A total of 79 DAT SPECT scans were included in this study (Fig. 3). SPECT was performed 3–4 h after intravenous injection of [^123^I]FP-CIT (mean amount 185 MBq, range 140–214 MBq). The patient characteristics can be found in Table 1.

**Table 1:** Demographic and clinical characteristics

	Patients (*n* = 79)
Age—years	63.4 ± 9.9
Male sex—number (%)	52 (65.8)
Symptom duration at imaging (mean ± SD)—years	1.8 ± 1.5
UPDRS[11] motor score at imaging (0–108) (mean ± SD)	18.2 ± 9.4

*SD* standard deviation, *UPDRS* Unified Parkinson’s Disease Rating Scale

### Analysis

[^123^I]FP-CIT SPECT imaging was performed in 30 hospitals (five tertiary referral hospitals and 25 community hospitals). Different dual-headed SPECT cameras and one brain-dedicated SPECT system were used. The initial assessment was “normal” in one patient and “abnormal” in 78 patients. The expert assessment was “normal” in two patients and “abnormal” in 77 patients. The concordance of the non-experts of the referring hospitals and JB on “normal” versus “abnormal” scans in this study was 98.7%.

### Use of DAT SPECT imaging survey

Seventy-six neurologists responded to the questionnaire (response rate 52%). 73.6% requested DAT SPECT imaging to differentiate between a neurodegenerative and non-neurodegenerative form of parkinsonism. 11.8% of the neurologists use DAT SPECT imaging to differentiate between PD and APS (Fig. 2).

## Discussion

Our findings are in line with the high diagnostic accuracy of visual assessments of DAT SPECT imaging in patients with a clinical diagnosis of early PD. In addition, we showed this is also true in routine clinical practice compared to a highly trained nuclear medicine physician dedicated to DAT imaging.

The strength of this study is that we evaluated the reliability of the visual assessments performed in clinical practice by nuclear physicians with various levels of expertise in neuroimaging in patients with a clinical diagnosis of early-stage PD. It was shown that false-positive (abnormal) outcomes occur more frequently among less experienced observers, while experienced observers classify [^123^I]FP-CIT SPECT scans correctly using visual assessment only [5]. Our findings suggest that this does not seem to be an issue in clinical practice, considering only one scan was found to be false positive by the expert. A limitation of this study is the selection bias. Our results reveal little about the accuracy of assessments of normal DAT SPECT scans considering the patients were already included in the LEAP study. The LEAP study population is not a typical population in that only patients were included in which the referring neurologist was fairly confident the patient had a diagnosis of PD. Consequently, the rate of abnormal DAT SPECT scans is considerably higher compared to routine clinical practice; therefore, our results may not be applicable to general use.

A possible limitation is that only one expert reassessed the images. Nevertheless, the inter-agreement of visual assessment of DAT SPECT imaging by experts, reported in the literature, is very high (Cohen’s κ 0.87–0.99) [10].

Furthermore, our survey showed that most of the responding neurologists request for [^123^I]FP-CIT SPECT imaging for an appropriate indication [3]. However, a significant portion (11.8%) of the responding neurologists request [^123^I]FP-CIT SPECT imaging to differentiate between PD and other APS, although DAT SPECT imaging cannot reliably discriminate between neurodegenerative parkinsonian disorders in routine clinical practice [4]. It is essential for clinicians who apply for [^123^I]FP-CIT SPECT imaging to have knowledge of the limitations and the information it can provide. With this survey, we gained a unique insight in the use of DAT SPECT imaging by neurologists in clinical practice.

## Conclusion

In conclusion, visual assessment of DAT SPECT imaging by non-experts in patients with a clinical diagnosis of early-stage PD is reliable. A significant portion of neurologists who request DAT SPECT scans is not always aware that the high accuracy is limited to the differentiation between neurodegenerative and non-neurodegenerative parkinsonism as DAT SPECT cannot reliably distinguish the various Parkinsonian syndromes.

## Data Availability

The datasets used and/or analyzed during the current study are available from the corresponding author on reasonable request.

## Abbreviations

[^123^I]FP-CIT: ^123^I-2β-carbomethoxy-3β-(4-iodophenyl)-N-(3-fluoropropyl) nortropane
APS: Atypical parkinsonian syndromes
DAT SPECT: Dopamine transporter single photon emission computed tomography
JB: Jan Booij
LEAP study: Levodopa in EArly Parkinson’s disease study
PD: Parkinson’s disease
